# Editorial: Psychosocial issues and interventions in pulmonary rehabilitation

**DOI:** 10.3389/fresc.2025.1562337

**Published:** 2025-02-12

**Authors:** Ingeborg Farver-Vestergaard, Eleonora Volpato, Henrik Hansen, Liz Steed

**Affiliations:** ^1^ Department of Medicine, Vejle Hospital, Vejle, Denmark; ^2^ Department of Regional Health Research, Faculty of Health Sciences, University of Southern Denmark, Odense, Denmark; ^3^ Department of Psychology, Università Cattolica del Sacro Cuore, Milan, Italy; ^4^ Heart-Respiratory Rehabilitation Unit, IRCCS Fondazione Don Carlo Gnocchi, Milan, Italy; ^5^ Research Group Health Psychology, University of Leuven, Leuven, Belgium; ^6^ Respiratory Research Unit and Department of Respiratory Medicine, Copenhagen University Hospital, Hvidovre, Denmark; ^7^ Institute of Rehabilitation Sciences, University of Antwerp, Antwerp, Belgium; ^8^ Institute of Clinical Medicine, University of Copenhagen, Copenhagen, Denmark; ^9^ Wolfson Institute of Population Health, Queen Mary University of London, London, United Kingdom

**Keywords:** chronic respiratory disease, behaviour change, psychological distress, holistic care, breathlessness

**Editorial on the Research Topic**
Psychosocial issues and interventions in pulmonary rehabilitation

Pulmonary rehabilitation is a cornerstone of care for individuals with chronic respiratory disease as it offers a structured approach to alleviate symptoms and improve physical function and quality of life (1–3). However, its effectiveness is deeply intertwined with a myriad of psychosocial and behavioural factors (4). In the context of chronic disease, rehabilitation programmes must encompass meaningful psychosocial interventions, that facilitate patient engagement in activities that contribute to maintaining their quality of life (5, 6). These efforts are most successful if they address the disease-related fear, psychological distress, fatigue and social isolation that respiratory conditions cause (7, 8). Understanding how such interventions can best be integrated into pulmonary rehabilitation to provide the optimal opportunity for holistic care based on the biopsychosocial approach to medicine, is important to guide practice.

A collection of articles in Frontiers of Rehabilitation Sciences addresses the psychosocial dimensions of pulmonary rehabilitation and highlights interventions that improve patient outcomes by addressing the interplay of psychological social and behavioural factors. To guide the discussion, we have organised these dimensions into four key themes:

1. Pulmonary rehabilitation as a dynamic, ongoing process
2. The role of psychosocial factors in symptom perception and management
3. Timing and tailoring of interventions to meet patient needs
4. Improving communication between patients and healthcare professionals

In the sections that follow, we consider each theme based on key findings from the featured articles. Finally, we identify potential implications for clinical practice and propose future directions for research and intervention development.

## Pulmonary rehabilitation as a dynamic, ongoing process

A unifying theme across the articles is the recognition of pulmonary rehabilitation as a continuous process rather than a finite program. Chronic respiratory diseases follow varied trajectories, requiring flexible approaches (9). For example, chronic obstructive pulmonary disease (COPD), as studied by Gronhaug et al., Farver-Vestergaard et al., Kaasgaard et al., involves a gradual decline punctuated by acute exacerbations. In contrast, lung cancer, as examined by Kaasgaard et al., often presents a more rapid and terminal progression. Meanwhile, lung transplant recipients, as explored by Song et al., face an intense post-transplant phase in which rehabilitation is crucial for recovery. These distinct trajectories underscore the need for multidisciplinary and adaptive care models that address shared and condition-specific challenges, such as breathlessness as studied by Pavy et al.

## The role of psychosocial factors in symptom perception and management

The studies in this collection underscore the significant role of psychological and social factors in shaping patients’ experience of their symptoms and their ability to manage them. Pavy et al.'s narrative review of nine experimental studies, in which breathlessness was induced in healthy individuals, demonstrates how unpredictable episodes of breathlessness can amplify fear and distress, thereby increasing the overall burden of these episodes. On the other hand, the predictability of such episodes has been shown to be associated with lower levels of distress. Similarly, Farver-Vestergaard et al.'s prospective study of individuals with COPD participating in a community-based pulmonary rehabilitation program emphasises how disease-specific anxiety management strategies, such as planning, problem solving and acceptance of physical symptoms, can be integrated into pulmonary rehabilitation activities and interactions with healthcare professionals and peers.

## Timing and tailoring interventions to meet patient needs

Another key theme is tailored interventions to address unique patient needs leading to better engagement and outcomes. For example, Kaasgaard et al. demonstrate that disease-specific rehabilitation programmes for patients with non-small cell lung cancer were associated with higher attendance rates and perceived relevance compared to generalised programs. Another study by Kaasgaard et al. indicates that self-reported activity levels and quality of life scores 5 years after initial PR were positively related to initial completion of the programme, regardless of whether patients had attended conventional physical exercise training or standardised group singing as the training modality.

The cross-sectional study by Song et al. identifies two distinct subgroups among stable lung transplant recipients up to 3 years post-transplantation: those with a high quality of life and those with a low quality of life. Their results indicate the importance of timely rehabilitation attention for the low quality of life-group, which was characterised by single lung transplantation, hospital readmission due to infection, low levels of optimism and mindfulness, and high levels of negative emotions. Identifying such characteristics could be facilitated by patient-reported outcome measures, as demonstrated in the study by Gronhaug et al., which will be discussed in the following section.

## Improving communication between patients and healthcare professionals

Effective rehabilitation depends on healthcare professionals understanding and addressing the unique needs and challenges of patients. In order to achieve this, communication between the patient and healthcare professional is essential, as the impact of symptoms and psychosocial issues cannot be objectively measured. Gronhaug et al. demonstrate how integrating patient-reported outcome measures (PROMs) into clinical practice facilitates meaningful dialogue and uncovers overlooked psychosocial challenges, enabling more personalised rehabilitation strategies.

## Implications and future directions

This collection advances our understanding of not only the content but also the approach to integrating psychosocial dimensions into pulmonary rehabilitation. Throughout, the importance of personalisation and multidisciplinary, patient-centred approaches is underscored. Table 1 summarises key findings from each theme listed in this editorial along with potential clinical implications and future directions.

**Table 1:** Key findings from the themes listed in the article collection.

Theme	Key findings	Implications and future directions
Pulmonary rehabilitation as a dynamic, ongoing process.	Different respiratory patient groups have different disease trajectories and needs.	Develop and test flexible, multidisciplinary models of care.
Psychosocial factors in symptom perception and management.	Psychosocial factors shape patients’ experience of symptoms, and targeting these factors can support symptom management.	Develop and evaluate integrated interventions designed to identify and address disease-specific fears, distress and coping mechanisms in the context of pulmonary rehabilitation.
Timing and tailoring interventions to patient needs.	Disease-specific programmes have the potential to improve engagement and outcomes in pulmonary rehabilitation, irrespective of the specific training modalities used.	Tailor interventions based on individual needs and preferences, follow-up on these needs over time and adjust if necessary.
Communication between patients and healthcare professionals.	PROMs facilitate dialogue and uncover hidden challenges when used optimally.	Develop guidelines and provide training for healthcare professionals on how to use PROMs to improve communication with patients.

PROM, patient reported outcome measures.

There are several areas that merit further exploration. First, current knowledge of biopsychosocial mechanisms is typically based on cross-sectional studies or studies with relatively short follow-up periods. As chronic respiratory diseases often develop over several years, longitudinal research is needed to capture the evolution of these factors over time. Second, the impact of respiratory disease extends to family carers, who often face unique emotional and practical challenges. It is likely that the role and burden of carers in chronic respiratory disease deviate from carer burden in other diseases, such as cancer, for which relatively more research is available. Third, distinguishing between disease-related psychosocial challenges and comorbid mental disorders in respiratory disease is complex. Further research is needed to develop accurate assessment methods and implement effective stepped-care intervention strategies.

## Conclusion

As respiratory diseases continue to pose significant global health challenges, addressing psychosocial issues at referral to and during pulmonary rehabilitation is essential. This research topic highlights and inspires the potential for pulmonary rehabilitation interventions that are as multidimensional and dynamic as the patients they serve.

## References

[1] Global Initiative for Chronic Obstructive Lung Disease. Global strategy for prevention, diagnosis and management of COPD: 2023 report. Global Initiative for Chronic Obstructive Lung Disease (2023). Available online at: https://goldcopd.org/2023-gold-report-2/ (Accessed February 06, 2025).

[2] Troosters T Blondeel A Janssens W Demeyer H. The past, present and future of pulmonary rehabilitation. Respirology. (2019) 24:830–7. 10.1111/resp.13517 30868699

[3] Spruit MA Singh SJ Garvey C Zu Wallack R Nici L Rochester An official American Thoracic Society/European Respiratory Society statement: key concepts and advances in pulmonary rehabilitation. Am J Respir Crit Care Med*.* (2013) 188:e13–64. 10.1164/rccm.201309-1634ST 24127811

[4] Holland A Dal Corso S Spruit MA. Pulmonary Rehabilitation (ERS Monograph). Sheffield: European Respiratory Society (2021).

[5] Lenferink A Lee AL. Education and self-management. In: Holland AE Dal Corso S Spruit MA, editors. Pulmonary Rehabilitation (ERS Monograph). Sheffield: European Respiratory Society (2021). p. 00–116.

[6] Farver-Vestergaard I Johannesen G Beek L ter. Occupational therapy, nutritional modulation and psychological support. In: Holland A Dal Corso S Spruit M, editors. Pulmonary Rehabilitation. Sheffield: European Respiratory Society (2021). pp. 83–98.

[7] Carl J Schultz K Janssens T von Leupoldt A Pfeifer K Geidl W. The “can do, do do” concept in individuals with chronic obstructive pulmonary disease: an exploration of psychological mechanisms. Respir Res*.* (2021) 22:1–10. 10.1186/s12931-021-01854-1 34615520 PMC8493747

[8] von Leupoldt A. Treating anxious expectations can improve dyspnoea in patients with COPD. Eur Respir J*.* (2017) 50:1701352. 10.1183/13993003.01352-2017 28899940

[9] Murray SA Kendall M Boyd K Sheikh A. Illness trajectories and palliative care. Br Med J. (2005) 330:1007–11. 10.1136/bmj.330.7498.1007 15860828 PMC557152

